# A minor alternative transcript of the fumarylacetoacetate hydrolase gene produces a protein despite being likely subjected to nonsense-mediated mRNA decay

**DOI:** 10.1186/1471-2199-6-1

**Published:** 2005-01-07

**Authors:** Natacha Dreumont, Antonella Maresca, Jean-François Boisclair-Lachance, Anne Bergeron, Robert M Tanguay

**Affiliations:** 1 Laboratory of Cellular and Developmental Genetics, CREFSIP, Dept Medicine, Université Laval, Pavillon Marchand, Ste-Foy, Québec, Canada G1K 7P4; 2 RNA Group/Groupe ARN, Département de Microbiologie et d'Infectiologie, Faculté de Médecine, Université de Sherbrooke, Sherbrooke, Québec, Canada J1H 5N4; 3 Stewart Biology Building, McGill University, Montreal, Quebec, Canada, H3A 1B1

## Abstract

**Background:**

Coupling of alternative splicing with nonsense-mediated mRNA decay (NMD) may regulate gene expression. We report here the identification of a nonsense alternative transcript of the fumarylacetoacetate hydrolase (*fah*) gene, which produces a protein despite the fact that it is subject to NMD.

**Results:**

During the characterization of the effects of the W262X nonsense mutation on FAH mRNA metabolism, two alternative transcripts (del100 and del231) of the *fah *gene were identified. Del100 lacks exon 8 and as a consequence, the reading frame is shifted and a premature termination codon appears at the 3'end of exon 10. Exons 8 and 9 are skipped in del231, without any disruption of the reading frame. Specific amplification of these transcripts demonstrate that they are produced through minor alternative splicing pathways, and that they are not caused by the W262X mutation *per se*. As shown with an antiserum raised against the C-terminal part of the putative DEL100 protein, the del100 transcript produces a protein, expressed at different levels in various human tissues. Interestingly, the del100 transcript seems to be subjected to nonsense-mediated mRNA decay, as its level was stabilized following a cycloheximide treatment.

**Conclusions:**

The del100 and del231 transcripts arise due to minor alternative splicing pathways and del100 is likely subjected to nonsense-mediated mRNA decay. However the remaining amount of transcript seems sufficient to produce a protein in different human tissues. This suggests that NMD has a broader role than simply eliminating aberrant transcripts and when coupled to alternative splicing, may act to modulate gene expression, by allowing the production of low amounts of protein.

## Background

Cells have evolved surveillance mechanisms to ensure the fidelity of gene expression. One such mechanism, nonsense-mediated mRNA decay (NMD), was discovered about twenty years ago in yeast [1] and then described in human inherited diseases caused by nonsense or frameshift mutations [2-4], which introduce premature termination codons (PTCs). Contrary to what would be predicted based on the nature of the mutation (a premature translational arrest), the resulting nonsense mRNAs rarely code for truncated protein products and are rather rapidly degraded [5]. Hence, NMD was first envisaged as a mean to protect cells against the effects of deleterious truncated proteins, with potential dominant-negative effects or a gain of function. Moreover, it seems that NMD has not solely evolved under the pressure of nonsense mRNAs originating from mutations but it also monitors PTC-containing transcripts arising from abnormalities in gene expression [6]. NMD plays a role in normal cellular development as examplified by the production of functional TcR and Ig genes. During lymphocyte maturation, these genes are subjected to extensive rearrangements and somatic mutation events. Approximately two-thirds of the rearranged genes are not in the proper translational reading frame and the resulting transcripts are down-regulated by NMD, ensuring that only functional TcR and Ig genes are expressed [7,8]. More recently, it was suggested that NMD could play a role in the regulation of gene expression. This was first suspected following the identification of unproductive splicing products of the SRp20 and SRp30b proteins or the ribosomal proteins L3, L7a, L10a and L12 in *Caenorhabditis elegans *[9,10]. The coupling of alternative splicing, which generates transcripts containing PTCs and NMD, which degrades these transcripts, enables the negative regulation of gene expression [9,10]. This system, termed RUST (for Regulated Unproductive Splicing and Translation, [11]) is also used in humans [12-14]. It was suggested that alternative splicing leading to NMD might prove to be a common mechanism of autoregulation of many splicing factors [12,15,16]. For example, the polypyrimidine tract binding protein (PTB), which generally acts as a splicing repressor, downregulates its own expression by repressing exon 11 inclusion in the mature mRNA [12]. The resulting alternative PTB mRNA lacking exon 11 contains a PTC and is subjected to NMD [12]. This negative autoregulation prevents the accumulation of PTB, and therefore the inappropriate processing of its targets [12]. This coupling of alternative splicing and NMD seems to be a rather common mechanism, as *in silico *analyses show that 35% of EST-suggested alternative transcripts contain PTCs [17]. Alternative splicing is thought to occur in 30–60% of human genes [18], and in addition to expanding proteome diversity, it may play a role in gene expression regulation by generating PTC-containing alternative isoforms. Interestingly, 10 to 30% of nonsense transcripts can escape NMD and are further immune to degradation [19]. Whether such transcripts code for proteins with a physiological function is unknown.

Fumarylacetoacetate hydrolase (FAH, E.C. 3.7.1.2) is the last enzyme of the tyrosine catabolic pathway. A deficiency in FAH causes hereditary tyrosinemia type I (HTI; OMIM 276700), the most severe disease of the pathway [20]. This inherited metabolic disorder is characterized by severe hepatic and renal dysfunctions often resulting in death in the first years of life if untreated. HTI displays phenotypic heterogeneity with both chronic and acute forms [20,21]. The *fah *coding gene located on chromosome 15 in the q23-q25 region [22] spans over 35 kb and contains 14 exons [23]. Forty-seven mutations have been identified so far in the *fah *gene, including 7 nonsense mutations [24-26]. While characterizing the effects of the W262X nonsense mutation on FAH mRNA metabolism, we identified two alternative transcripts, del100 and del231 in a HTI patient homozygous for W262X. These transcripts are found in normal cells and thus are not due to the nonsense mutation *per se*. Interestingly, del100 has skipped exon 8 and as a consequence, the reading frame is shifted, with the appearance of several new PTCs. This transcript is therefore likely subjected to NMD, as suggested by a block of translation by cycloheximide. However, the amount of nonsense transcript which escapes NMD seems to be sufficient to produce a protein of 31-kDa, detected in several human tissues. This report suggests that NMD may allow for the production of low amounts of protein.

## Results

### Identification of two alternative transcripts of the *fah *gene

**Figure 1:**
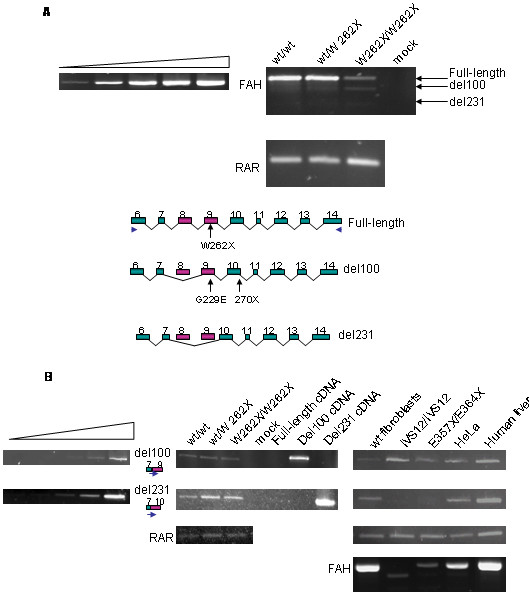
**Del100 and del231 result from minor alternative splicing pathways and not from the presence of the W262X mutation. **Total RNA extracted from different normal cell lines or cells of HTI patients was subjected to RT-PCR. Controls for FAH, del100 and del231 are displayed on the left to show that the PCR reactions are in the linear range. (A) FAH amplification from exons 6 to 14 in the lymphoblastoid cell lines. The two additional products (del100 and del231) seen in the homozygous cell line (W262X/W262X) are depicted below the gels. Exons are represented by boxes and introns by lines. Del100 has skipped exon 8 and as a consequence, the reading frame is shifted (W262X becomes G229E). A first PTC is located at the 3'end of exon 10 (270X). Del231 has skipped both exons 8 and 9, but without any change in the reading frame. (B) Primers designed to specifically amplify del100 and del231 span exon 7 to 9 or exon 7 to 10 junctions respectively (depicted in blue). Full-length, del100 or del231 cDNAs were cloned in pRC/CMV and were amplified to verify primer specificity to each alternative transcript. Both transcripts are present in normal cells (wild-type lymphoblasts or fibroblasts and HeLa cells) and human liver. Del100 is amplified in HTI cells with a splice mutation in exon 12 (IVS12/ IVS12) or two nonsense mutations in exon 13 (E357X/E364X). FAH was also amplified as a control in the different human cell lines.

The W262X mutation is a G->A transversion located in exon 9 of the *fah *gene at nucleotide position 786 and is frequent in the Finnish population [27]. No protein was detected in the liver or the fibroblasts of homozygous patients [28]. Consistent with this type of mutation, we demonstrated that W262X mRNAs are degraded by NMD in the cytoplasm [29]. While studying the decay of W262X nonsense FAH transcripts in lymphoblastoid cell lines, we repeatedly observed two additional RT-PCR products in homozygous W262X/W262X cells (Figure 1A). Purification and sequence analysis of these two products revealed that they were alternative transcripts of the *fah *gene. The first one, del100, lacks exon 8 (Figure 1A) and the second one, del231, which is less abundant, lacks both exons 8 and 9 (Figure 1A). In del100, the skipping of exon 8 from the mature transcript causes a shift in the reading frame and as a consequence, several new PTCs appear, the first one being located at the 3' end of exon 10, at the new amino acid position 270 (Figure 1A). The G786A mutation does not code for a stop codon in del100 but causes the replacement of a glycine by a glutamate residue (Figure 1A). Del231 does not show further disruption of the open reading frame downstream of the deletion and is predicted to code for a shorter FAH-like protein (about 34-kDa) missing the region encoded by exons 8 and 9 (Figure 1A).

Del100 and del231 were first identified in the liver of a patient harboring another mutation (Q279R), which weakens the donor splice site of exon 9 [30]. Because the W262X mutation is located in the same exon and that in some cases a nonsense codon can affect splicing [31], we wondered whether these two transcripts were due to an effect of the W262X mutation on a cis-acting splicing element in exon 9. To test this hypothesis, RT-PCRs were performed using primers spanning the exon7-exon9 junction or the exon7-exon10 junction to specifically amplify del100 and del231 respectively. As shown in Figure 1B, both del100 and del231 transcripts were detected by this method, in homozygous mutant cells (W262X/W262X; Figure 1B, middle panel), as well as in normal cells (wt/wt; Figure 1B, middle panel). The identity of these amplification products was verified by sequencing (data not shown). A similar result was found in various human cell lines (Figure 1B, right panel). Indeed, del100 and del231 were amplified in fibroblasts, HeLa cells (Figure 1B, right panel) and in human liver (Figure 1B, right panel), the tissue where FAH is the most expressed [32]. Moreover, del100 was amplified in two HTI cell lines (Figure 1B), which harbor either a splice mutation in intron 12 (IVS12/IVS12; [33]) or two nonsense mutations in exon 13 (E357X/E364X; [33]). Del231 was not detected in these two cell lines as expected, since these mutations introduce PTCs either following exon 12 skipping (IVS12/IVS12) or due to the two nonsense mutations themselves (E357X and E364X), that likely target the nonsense transcripts to the NMD pathway.

Altogether, these data strongly argue in favor of del100 and del231 being minor alternative transcripts of the *fah *gene, rather than resulting from the presence of the W262X mutation.

### The del100 transcript is translated into a protein

**Figure 2:**
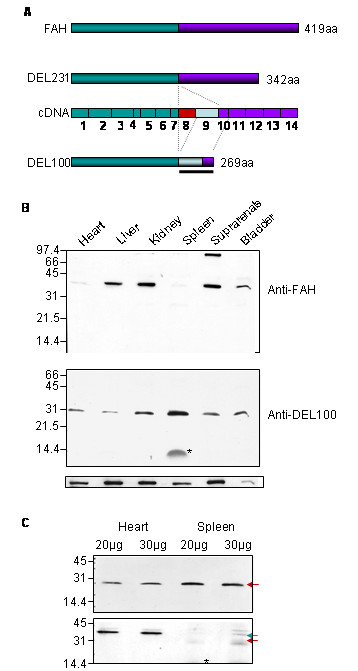
**Del100 transcript encodes for a protein expressed in various tissues. **(A) Schematic representation of the putative DEL100 and DEL231 proteins. FAH and DEL231 proteins are identical, except for the region encoded by exons 8 and 9, which is missing in DEL231. DEL100, due to the skipping of exon 8 and the resulting change in the coding frame, differs from FAH at its C-terminal end. The black bar below the DEL100 protein represents the region used to raise the antiserum. (B) 30 μg proteins of a 10% (w/v) tissue homogenate were separated on a 15% SDS-polyacrylamide gel. Proteins were transferred to a nitrocellulose membrane and blotted using a rabbit anti-FAH (top panel; dilution 1:25,000) or a mouse anti-DEL100 antiserum (middle panel; dilution 1:1,000). A band with an apparent molecular weight of 31-kDa is detected by the anti-DEL100 antiserum in all tissues tested. The star indicates non-specific signal. The membrane was blotted against β-actin to control for protein loading. (C) The 31-kDa band (indicated by a red arrow) was detected in spleen using a monoclonal antibody directed against the N-terminal part of the FAH (green arrow).

The identification of two minor alternative transcripts of the *fah *gene raised the question whether they resulted from errors of the splicing apparatus and were unproductive alternative transcripts or whether they could produce protein products with potential physiological roles. There is presently no reported indication for the existence of additional FAH isoforms. The DEL231 open reading frame is identical to FAH, except for the missing region encoded by the skipped exons 8 and 9 and corresponding to amino acids 203 to 280 (Figure 2A). The open reading frame of the del100 transcript is identical to FAH and del231 from the ATG start codon to amino acid 202. However, the last 67 amino acids of the putative DEL100 protein encoded by exons 9 and 10 are completely different, due to the shift in the reading frame following exon 8 skipping (Figure 2A). To find out if del100 was translated into a protein, we raised an antiserum against the last 67 amino acids of the putative DEL100 protein and used it to search for the presence of this protein in different adult human tissues. These tissues were obtained after an autopsy and only one sample per tissue was tested, due to the difficulty to obtain them. As shown in Figure 2B (middle panel), a cross-reacting band was present in heart, liver, kidney, spleen, suprarenals and bladder. The DEL100 protein has an apparent molecular weight of 31-kDa, consistent with the value of 29.7-kDa calculated from its sequence. Interestingly, the expression level of the DEL100 protein varied between the different tissues and it differed from that of FAH (Figure 2B, top panel). For example, FAH was barely detected in the spleen, whereas the expression level of the DEL100 protein was the highest in this tissue (Figure 2B). A monoclonal antibody directed against the N-terminal part of the FAH protein was used to detect both FAH and DEL100 in the tissues where FAH is the less expressed (Figure 2C). DEL100 was barely detected in the spleen, suggesting that the protein is synthezised in very low amounts.

**Figure 3:**
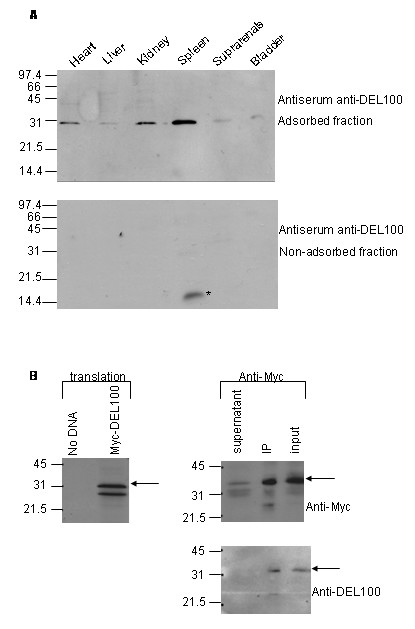
**Specificity of the anti-DEL100 antiserum. **(A) The mouse antiserum was affinity purified on the recombinant C-terminal of DEL100 and used to probe the same membrane as in Figure 2B. The adsorbed fraction (top panel), specific of the C-terminal of DEL100, still recognizes the 31-kDa protein. The non-adsorbed fraction (bottom panel) shows no cross-reactivity to the protein. The star indicates a non-specific signal. (B) The DEL100-Myc protein was synthesized in a rabbit reticulocyte lysate and labeled with ^35^S (translation panel). The tagged protein is indicated by an arrow. The band below is the protein synthesized without the Myc tag. The anti-Myc was immobilized on protein A-sepharose and incubated with *in vitro *translated DEL100-Myc. Effectiveness of the immunoprecipitation was verified by blotting using the anti-Myc (1/2,000) antibody. The anti-DEL100 antiserum (1/1,000) recognizes the *in vitro *translated protein in the input (10% of the reaction) and the immunoprecipitate (IP, indicated by an arrow).

The specificity of the signal was verified by adsorbing the antiserum on the purified C-terminus of DEL100, used for the mouse immunization. As shown in Figure 3A (top panel), the affinity-purified antiserum still recognized the 31-kDa protein, whereas the non-adsorbed fraction did not show any cross-reactivity (Figure 3A, bottom panel). The protein of low molecular weight, which is recognized by the non-adsorbed antiserum in the spleen, is unspecific background (Figure 2B, middle panel and Figure 3A, bottom panel; indicated by a star). A DEL100 protein with the Myc tag was synthesized in an *in vitro *transcription-translation assay and immunoprecipitated using an anti-Myc antibody. The anti-DEL100 antiserum recognizes the immunoprecipitated DEL100-Myc protein, further demonstrating its specificity (Figure 3B). Altogether these results suggest that the del100 alternative transcript is translated into a protein of 31-kDa whose expression in different tissues differs from that of FAH.

### The del100 transcript seems to be subjected to NMD

**Figure 4:**
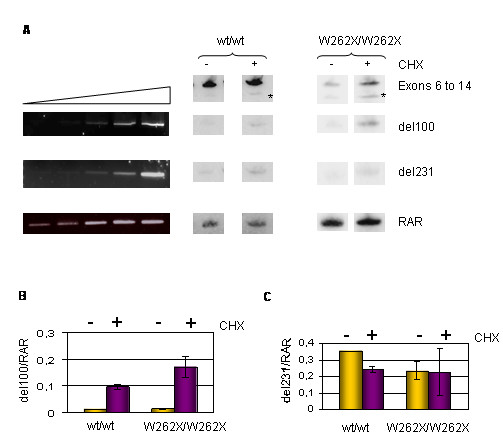
**Del100 is subjected to NMD. **Control cells (wt/wt) and the homozygous cell line from the Finnish patient (W262X/W262X) were treated with 100 μg/ml cycloheximide (CHX) for 3 hours. Total RNA was extracted and subjected to RT-PCR. RAR serves as a control for RNA quantity in each sample. Control amplifications (showing an increasing number of cycles) for del100, del231 and RAR are displayed on the left to show that, with the conditions used, each PCR reaction is in the exponential phase. (A) [α^33^P]-dATP was incorporated during the PCR reaction and the products loaded on a 6% acrylamide gel. The gel was used to directly expose an X-ray film and the signal was quantified using the NIH Image 1.2 software. FAH was amplified from exons 6 to 14 (the del100 transcript which is detected using these primers is indicated by a star). Del100 and del231 were amplified using the specific primers RT84 and RT85. (B) Quantification of the amount of del100 mRNA in 3 different experiments, normalized to RAR levels. The error bars represent standard deviations. Del100 is stabilized between 4- to 7-fold in homozygous cells (W262X/W262X) and normal cells (wt/wt) following the cycloheximide treatment (yellow bars: - cycloheximide; purple bars: + cycloheximide). (C) del231 is relatively unaffected by translation inhibition.

The skipping of exon 8 in del100 causes a change in the reading frame and as a consequence, several new stop codons appear (different from the W262X mutation). To verify if the nonsense del100 transcript was subjected to NMD, lymphoblastoid cells were treated with cycloheximide (Figure 4) an inhibitor of translation. Stabilization of nonsense transcripts following such a treatment suggests that they are degraded through the NMD pathway [34]. The effectiveness of the treatment was previously verified on the full-length W262X containing transcript [29] and an example is given in Figure 4A (upper panel). The full-length transcript was up-regulated in the homozygous cell line (Figure 4A; W262X/W262X) but remains unaffected in wild-type cells (Figure 4A; wt/wt). The same treatment was used to determine the fate of the del100 transcript (Figure 4A and 4B). A stabilization of this alternative transcript was observed when the FAH transcripts were amplified from exons 6 to 14 in wild-type and homozygous cells (indicated by a star in Figure 4A). The same result was obtained using a specific amplification of the del100 transcript (Figure 4A, second panel and Figure 4B). The amount of del100 following cycloheximide treatment increased about 5-fold the level observed in untreated cells (Figure 4B). These results suggested that del100 is indeed subjected to NMD in homozygous cells and in normal cells as well. In contrast del231, which does not contain any PTC as a result of the skipping of both exons 8 and 9, seemed relatively unaffected by the cycloheximide treatment as expected (Figure 4C) and does not seem to be subjected to NMD. This result confirmed those obtained in E357X/E364X or IVS12/IVS12 HTI fibroblasts (Figure 1B), where del231 was undetectable probably because of the introduction of PTCs in these transcripts and their targeting to the NMD pathway.

## Discussion

**Figure 5:**
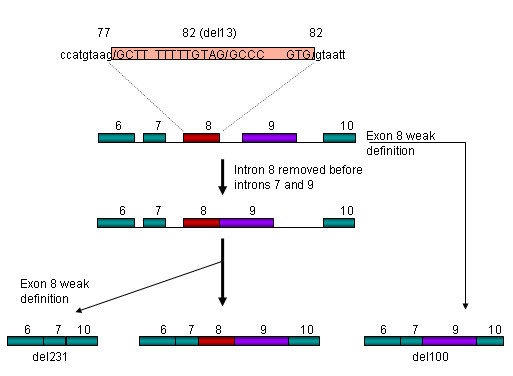
**Suspected splicing pathways of the region encompassing exons 6 to 10. **Schematic representation of exon 8 and its splice sites is given on the top of the figure. The effectiveness of the donor and acceptor splice sites were determined using the webgene program  and is indicated above the sequence. A cryptic acceptor splice site is used in the case of the IVS6-1g->t mutation (described in [35]). Exons are represented by boxes and introns by lines. The thick arrow is the major splicing pathway, whereas thin arrows are minor alternative splicing pathways. We suggest that del231 could be explained by the fact that intron 8 is spliced out before introns 7 and 9, leading to an intermediate in which exons 8 and 9 are defined together. Because of exon 8 weak definition, a minority of transcripts eliminates both exons 8 and 9. Del100 could be produced by another minor splicing pathway, in which exon 8 is skipped alone.

Del100 and del231 were originally identified while studying the impact of NMD on hereditary tyrosinemia type I. During the characterization of the effects of the W262X mutation on FAH mRNA metabolism [29], we detected two minor alternative transcripts. If due to the W262X mutation, they should only be produced when the nonsense mutation is present, i.e. in heterozygous (W262X/wt) and homozygous (W262X/W262X) cell lines. However, by using specific primers for each transcript, we found that both del100 and del231 are produced in normal lymphoblastoid cells (Figure 1B) and in normal human liver. Del100 was also present in two different HTI cell lines harboring different nonsense mutations (E357X/E364X) or a splice mutation (IVS12+5g->a) in exon 13 or intron 12 respectively. These data argue in favor of del100 and del231 resulting from alternative splicing pathways rather than from a W262X-associated altered splicing mechanism. We suggest that both transcripts result from a weak definition of exon 8 (Figure 5). Indeed, exon 8 is subjected to many alterations as a result of splice mutations in the region encompassing exons 6 to 9. For example, due to a splice mutation in intron 6 (IVS6-1g->t; [30,35]), a cryptic acceptor site in exon 8 is activated or exon 8 is skipped [30,35]. Del100 and del231 were also identified in the case of the Q279R mutation, a splicing mutation that weakens the donor splice site of exon 9 [30]. Figure 5 presents with a model that could explain these observations: we suggest that in the major splicing pathway, intron 8 is removed before introns 7 and 9, leading to a splice intermediate that contains the merged exons 8 and 9. Both exons are subsequently defined as a single exon. The order of intron removal is an important determinant of the outcome of splice-site mutations and could explain some unusual alterations, like the skipping of contiguous exons, as strongly suggested by studies of the COL1A1 and COL5A1 splice mutations [36,37]. Altogether, these data suggest that at least del231 may arise through a minor splicing pathway due to an error-prone splicing apparatus because of the weak definition of exon 8 and the order of intron removal. Del100 could originate from a second minor splicing pathway, in which exon 8 is skipped alone because of its weak definition (Figure 5).

Del100 and del231 are thus the first cases of alternative splicing for the *fah *gene. However, it remained to see whether they were unproductive splice isoforms or whether they could code for protein isoforms. The del231 transcript retains an unchanged open reading frame when compared to FAH. The putative DEL231 protein would be similar to FAH except for the lower molecular weight (about 35-kDa), due to the missing region encoded by exons 8 and 9. We have been unable to detect a protein species of the size that could correspond to DEL231 using an antibody against full-length FAH. Whether this reflects the absence of such a protein or its presence in a very low amount undetectable with the presently available antibodies remains unknown. The latter explanation seems plausible since the del231 transcript, although not subjected to NMD, is much less abundant that the full-length FAH transcript or del100, as it is barely detected in W262X cells with the RT76 and RT025 primers (Figure 1) and the number of PCR cycles needed for its visualization is higher than for del100 when using the specific primers.

The structure of the putative DEL100 protein in the N-terminal part is identical to that of FAH. But due to exon 8 skipping, the reading frame is very different in the last 67 amino acids. DEL100, a 31-kDa protein, was detected in different human tissues using an antiserum raised against the specific C-terminal part of the putative protein. The antiserum is specific for the DEL100 protein and does not cross-react with FAH. In addition, the cross-reacting 31-kDa band was lost after adsorbing the antiserum against the purified peptide used for the immunization and the antiserum recognizes an *in vitro *translated DEL100-Myc protein, demonstrating its specificity. Thus the del100 transcript seems to direct the synthesis of a protein. This result is surprising because this transcript contains PTCs and seems to be subjected to NMD, as shown by a block of translation following a cycloheximide treatment. Interestingly, FAH and DEL100 have converse expression patterns in the human tissues examined. This suggests a post-transcriptional regulation of the expression of the two proteins, since the two transcripts originate from the same pre-mRNA. Alternative splicing, a highly regulated process, which can be developmental-stage or cell-specific, could be responsible for this difference of expression. For example, exon 8 may be more prone to skipping in the spleen given the concentration of specific *trans*-acting splicing regulators. This could be a way to downregulate the level of FAH transcript, by producing an alternative transcript, which is further eliminated by NMD. Indeed recent *in silico *analyses and observations on splicing factors have suggested that NMD, when coupled to alternative splicing, could regulate gene expression [10-12,17]. Del100 could be another example of such a coupling of alternative splicing and NMD. In such a case, DEL100 would not be expected to play any function in the cell.

Very low levels of proteins can sometimes have enormous effects. Interestingly, 10–30% of nonsense transcripts escape NMD and when associated with polysomes are stable [19]. Is the coupling of alternative splicing with NMD in order to degrade the unproductive isoform the only option? An alternative, as proposed by Neu-Yilik *et al*. [38], may be that NMD could function in quantitatively controlling the expression of low amounts of protein. In this view, the PTC-containing del100 transcript may produce a protein with a physiological, although still unknown, function in the cell. The FAH structure contains a C-terminal part of 300 residues, which presents a novel arrangement of β-strands and plays a functional role in Ca^2+ ^binding, dimerization and catalysis of its substrate, fumarylacetoacetate [39]. Many of the residues encoded by exon 8 are part of the β-strands and residue 233 serves to bind the Ca^2+ ^[39]. The DEL100 protein, which lacks these residues, is thus very unlikely to function in catalyzing the hydrolytic cleavage of carbon-carbon bonds. While the function of DEL100 is unknown at this time, it may have a function in tyrosinemia. Indeed, not all mutations affecting the *fah *gene will similarly affect the DEL100 protein. For example, mutations affecting exons downstream of exon 10 will affect FAH production but not that of the DEL100 protein. This might be reflected in the phenotypic heterogeneity observed in HTI patients [21]. Preliminary computer analyses of DEL100 motifs using Proscan at PBIL suggest that it contains a putative DNA-binding motif (RVFLQNLLSvSQARLR with 89% similarity found to the consensus sequence). Whether DEL100 can function as a regulating factor remains unknown.

## Conclusions

NMD was first envisaged as a mechanism to prevent the accumulation of faulty transcripts, arising from mutations or processing abnormalities. Recent *in vivo *observations and *in silico *analyses have suggested a new role of NMD in gene expression regulation, when coupled to alternative splicing. We report here the identification of an alternative nonsense transcript of the *fah *gene, which despite being subjected to NMD, produces a protein in different human tissues. This provides an interesting starting point for the analysis of the role of NMD in the regulated productive splicing and translation.

## Methods

### Cell culture

The lymphoblastoid cell lines were established from lymphocytes of a HTI patient and his parents as described in Tremblay and Khandjian [40]. Cells were maintained in RPMI-1640 supplemented with 15% fetal bovine serum. The other human cell lines, HeLa (cervix) and normal fibroblasts, were cultured in DMEM 10%. Fibroblasts of other HTI patients (WG1647, mutation IVS12/IVS12; and WG1922, mutation E357X/E364X [33]) obtained from the Montreal Children's Hospital (C.R. Scriver) were maintained in DMEM 10% FBS. In translation inhibition experiments, lymphoblastoid cells were treated with 100 μg/ml cycloheximide 3 hours prior to RNA extraction (see below).

### RT-PCR analysis

Total RNA was extracted from 5·10^6 ^cells with Trizol reagent (Gibco-BRL). RNA from human normal liver was extracted using the RNAqueous kit (Ambion). 1 μg RNA was reverse transcribed using an oligo(dT) and Stratascript (Stratagene). FAH cDNA was amplified from exons 6 to 14 using the following primers: RT76 (5'-CGT GCC TCC TCT GTC GTG-3') and RT025 (5'-GGG AAT TCT GTC ACT GAA TGG CGG AC-3'). Sense primers were designed to specifically amplify del100 and del231. RT84 (5'-TGG AGC TGG AAA TGC ACG-3') spans the exon 7 to exon 9 junction, whereas RT85 (5'-TGG AGC TGG AAA TGG ACC-3') spans the exon 7 to exon 10 junction. Amplification of the alternative transcripts with RT84 or RT85 was performed using HotStart (Qiagen) and PCR conditions were optimized in order to minimize nonspecific hybridization of the primers. Moreover, for each amplification (the FAH transcripts and RAR), the kinetic of the reactions were performed and the number of cycles used for each PCR was in the exponential phase.

Analysis of the cycloheximide treatment was done as previously described [29].

### Production of an antiserum against the DEL100 protein

An antiserum against the C-terminal part of the DEL100 protein was raised in mouse. The antigen is the C-terminal part (the last 67 amino acids) of the DEL100 protein and is different from FAH or the DEL231 protein. FAH cDNA was amplified from exon 9 to exon 14 using the primers hFAHsstermdel100 (5'-CGG GAT CCC TGC AGC ACG AGA CAT TCA GAA GTG G-3') and RT025 with Expand High Fidelity. The PCR product was inserted into pET30a (Novagen) at the BamHI and EcoRI sites, in order to express the reading frame of the C-terminal part of the DEL100 protein. The His-Tag fusion protein used for immunization was purified by affinity chromatography on a Ni-NTA column (Qiagen) in denaturing conditions with 6 M urea.

### Preparation of the anti-hFAH monoclonal antibody

The anti-hFAH monoclonal antibody was raised against the N-terminus of the protein. The 161 residue peptide was obtained by cutting the pET30a-FAH vector [41] with the NcoI and XhoI restriction enzymes (New England Biolabs), overhangs were then filled using T4 DNA polymerase (New England Biolabs) and the vector was ligated with the T4 DNA ligase (New England Biolabs). The peptide was expressed in the GJ1158 strain of *Escherichia coli *as previously described [41] and purified by affinity chromatography on a Ni-NTA column under denaturing conditions (6 M urea). The purified peptide was injected into BALB/c mice and hybridomas were prepared according to the procedures described in [42].

### Western blot analysis

Cells were harvested and lysed in 1 × SDS sample buffer (62.5 mM Tris-Hcl, pH 6.8, 2% SDS, 2.5% 2-β mercaptoethanol, 75 mM DTT, 10% glycerol and 0.005% Bromophenol blue). Human tissues obtained at autopsy and stored at -70°C until used [32] were homogenized in 10% (w/v) 0.01 M K-phosphate buffer (pH 7.3) and centrifuged for 20 min at 15,000 g. The supernatant was used for immunoblot assay. Samples were electrophoresed on SDS-15% polyacrylamide gels and proteins transferred to a nitrocellulose membrane. Antiserum against the DEL100 protein was used at a dilution of 1:20,000 against purified proteins or 1:1,000 against human tissues. FAH was detected using the polyclonal antibody #488 (1:25,000) as described previously [32] or using a monoclonal antibody directed against the N-terminal part of the FAH protein (dilution 1/500). Protein loading was verified by using a monoclonal antibody against β-actin (dilution 1/400; Neomarker).

### Tests of the specificity of the anti-DEL100 antiserum

In some experiments, the mouse antiserum against the DEL100 protein was adsorbed on the recombinant His-tag C-terminal protein blotted on nitrocellulose. After an overnight incubation at 4°C, the non-adsorbed fraction was removed and conserved for further characterization. The adsorbed antibody fraction (affinity purified) was eluted using 1 ml of glycine-HCl 0.1 M, pH 2.8 and the pH immediately neutralized by adding 100 μl of 1 M K_2_HPO_4_, pH 8.2.

The del100 cDNA was obtained by RT-PCR on total RNA extracted from W262X/W262X cells using ND1 (5' CCC AAG CTT CAG CAT GTC CTT CAT CCC GGT GG 3') and ND2 (5' TGC TCT AGA TTT ATT TGT CAC TGA ATG GCG G 3'). The amplification products were cloned into pDrive (Qiagen) and different clones were sequenced. One clone containing the del100 cDNA was used for further cloning. It was amplified using 5'del100-Eco (5' GGA ATT CCA GCA TGT CCT TCA TCC 3') and ND2. The amplified fragment was digested with EcoRI and XbaI and ligated into EcoRI-XbaI-digested pcDNA3-myc RANGAP, replacing the insert coding for RANGAP. pcDNA3-mycRANGAP was kindly provided by Dr M. J. Matunis (Johns Hopkins University, Baltimore, MD) The construct was used for coupled *in vitro *transcription-translation using the TNT coupled reticulocyte lysate system (Promega) according to the manufacturer's recommendations. 25 μl of the reaction were used for immunoprecipitation using the anti-Myc antibody as follows: the anti-Myc (1/100) was incubated 1 hour with protein A-sepharose beads (Sigma). The antibody was next immobilized on the beads using 20 mM dimethyl pimedilate (Sigma) in 0.2 M borate sodium (pH 9.0) for 30 min at room temperature. The reaction was stopped by washing the beads twice in 0.2 M ethanolamine and incubation in this solution for 2 hours at room temperature. The antigen (25 μl of the *in vitro *translated DEL100-Myc protein) was incubated with the beads for two hours at 4°C in a dilution buffer containing 10 mM Tris-HCl pH 8.0, 1 mM EDTA and 10% glycerol. The immunoprecipitated protein was eluted by adding 25 μl of SDS loading buffer (62.5 mM Tris-HCl, pH 6.8; 2% SDS; 2.5% β-mercaptoethanol; 75 mM DTT; 10% glycerol and 0.01% bromophenol blue). Samples were electrophoresed on SDS-15% polyacrylamide gels and proteins transferred to a nitrocellulose membrane. The anti-Myc was used at a dilution of 1/2,000 and the anti-DEL100 antiserum at a dilution of 1/1,000.

## List of abbreviations

CHX, cycloheximide; ESE, exonic splicing enhancer; FAH, fumarylacetoacetate hydrolase; HTI, hereditary tyrosinemia type I; Ig, immunoglobulin; NMD, nonsense-mediated mRNA decay; PCR, polymerase chain reaction; PTB, polypyrimidine tract binding protein; PTC, premature termination codon; RAR, retinoic acid receptor; RT, reverse transcription; RUST, regulated unproductive splicing and translation; TcR, T-cell receptor.

## Author's contributions

ND carried out the experiments and wrote the manuscript. AM participated in the design of the study. JFBL participated in the cloning of Del100 into pcDNA3. AB raised the mAb directed against the N-terminal part of the FAH protein. RMT participated in the design, coordination of the study, and in the writing of the manuscript.
